# Assessing cloud QoS predictions using OWA in neural network methods

**DOI:** 10.1007/s00521-022-07297-z

**Published:** 2022-05-14

**Authors:** Walayat Hussain, Honghao Gao, Muhammad Raheel Raza, Fethi A. Rabhi, Jose M. Merigó

**Affiliations:** 1 Victoria University Business School, Victoria University, Melbourne, VIC 3000 Australia; 2 School of Computer Engineering and Science, Shanghai University, Shanghai, 200444 China; 3 Department of Software Engineering, Firat University, Elazig, Turkey; 4 School of Computer Science and Engineering, University of New South Wales, Sydney, NSW 2052 Australia; 5 Faculty of Engineering and Information Technology, University of Technology Sydney, Sydney, Australia

**Keywords:** Computational complexity, Time-series forecasting, Cloud QoS, Deep neural network, Complex prediction, OWA, Service level agreement

## Abstract

Quality of Service (QoS) is the key parameter to measure the overall performance of service-oriented applications. In a myriad of web services, the QoS data has multiple highly sparse and enormous dimensions. It is a great challenge to reduce computational complexity by reducing data dimensions without losing information to predict QoS for future intervals. This paper uses an Induced Ordered Weighted Average (IOWA) layer in the prediction layer to lessen the size of a dataset and analyse the prediction accuracy of cloud QoS data. The approach enables stakeholders to manage extensive QoS data better and handle complex nonlinear predictions. The paper evaluates the cloud QoS prediction using an IOWA operator with nine neural network methods—Cascade-forward backpropagation, Elman backpropagation, Feedforward backpropagation, Generalised regression, NARX, Layer recurrent, LSTM, GRU and LSTM-GRU. The paper compares results using RMSE, MAE, and MAPE to measure prediction accuracy as a benchmark. A total of 2016 QoS data are extracted from Amazon EC2 US-West instance to predict future 96 intervals. The analysis results show that the approach significantly decreases the data size by 66%, from 2016 to 672 records with improved or equal accuracy. The case study demonstrates the approach's effectiveness while handling complexity, reducing data dimension with better prediction accuracy.

## Introduction

Cloud computing has a dynamic and uncertain nature in which a consumer can request services based on their business demand [1]. The uncertainty makes it pivotal for the service provider to proactively manage the risk of possible Service Level Agreement (SLA) violations [2]. Quality of Service (QoS) is the primary indicator to measure the performance of service-oriented applications. QoS illustrates the functional and non-functional attributes of services that are encapsulated within a Service Level Agreement (SLA) formed between a consumer and the provider. Implementation of these QoS parameters, such as security, availability, reusability, and others, ensures adequate service quality and management, resulting in a trusted relationship among stakeholders [3]. The service provider has an SLA breach when it fails to meet the promised target for agreed SLA metrics. The service provider is liable for SLA violation penalties, including service credit, penalty fees, licence extension, and support. The SLA violation influences the reputation and trust of the service provider, which could impact potential new consumers. One possible way to mitigate the risk of SLA violation is the QoS prediction.

Several approaches have tried to predict QoS parameters to avoid SLA violation optimally. Most of these approaches adopted collaborative filtering methods [4–6] to find the relationship between services and consumers [7]. Li et al. [4] proposed a time-aware cloud service recommendation algorithm based on a Time-aware Matrix Factorisation (TMF) model for QoS prediction. The approach used a collaborative filtering method in matrix factorisation to predict QoS parameters. The predicted results are then passed to a temporal smoothing method to obtain final-time aware QoS predictions for service recommendations. Hussain et al. [5] used a user-based and item-based collaborative filtering method with an enhanced K-NN algorithm to predict future QoS parameters to avoid SLA violation. Discussed approaches tried to find optimal QoS prediction using user-based or item-based filtering methods. The approaches attempted to make an informed decision for SLA violation; however, they could not accommodate complex QoS predictions. The approaches were unable to prioritise a particular set of QoS parameters over another. Nagarajan and Thirunavukarasu [8] proposed a service context-aware cloud broker method in another approach which pulls service features from cloud services using relevant data and evaluates service similarity using QoS parameters. The matrix factorisation concept addresses the cold start problem and forecasts higher QoS values for arriving customers. Shadabfar et al. [9] proposed a susceptible-exposed-infected-vaccinated-recovered (SEIVR) model to predict the spread of COVID-19. The authors considered multiple variables – transmission, recovery, and mortality. Sioofy Khoojine et al. [10] proposed an autoregressive network model to predict COVID-19 disease in another method. The discussed approaches work well in different problems, including healthcare, cloud and web services. However, in a complex nonlinear service-oriented framework where the QoS parameters are distributed widely across the distributed network, it is very difficult for the decision-maker to prioritise a certain set of data to make a complex prediction.

Machine learning (ML) algorithms are widely used in predictive models that allow a complex nonlinear relationship between responsive variables and predictors. Neural networks are data-driven algorithms that learn patterns from the dataset [11]. The main issue for different supervised learning algorithms is the specific requirements. To get optimal prediction results, the training dataset needs to be pretty good with a low avoidable bias. Furthermore, the training set needs to generalise very well to the development set. Besides that, it has been observed that the computational complexity significantly increases with an increase in a training dataset [12]. Different gradient descent optimisation techniques such as RMSProp, Adamax [13], and Adam are commonly used to address computational complexity problems. Deep autoencoder [14] is used to reduce the dimensionality of the input in a labelling layer [15, 16]. Most machine learning methods have convergence issues towards the global minimum. Moreover, it is challenging to manage high-order datasets of varying time intervals, such as QoS data. Like the traditional prediction methods, ML algorithms do not have any mechanism to assign variable weights to different intervals and reduce data dimensions without losing any information. Furthermore, the computational complexity increases with an increase in data dimensions [12].

Computational complexity defines the number of computational resources required to solve a specific problem by systematic algorithm application [17]. Computational complexity is commonly categorised into time and space and ordered into P, NP, NP-complete and NP-hard [18] problems. Different approaches try to address the issue, such as Scutari, Vitolo and Tucker [19] analysed the time complexity of Bayesian network structure with the greedy search. The study found that considering closed-form estimators for local distribution with few parents can significantly impact the complexity of a network. In another experiment, Alizadeh, Allen and Mistree [20] found that Multivariate Adaptive Regression Splines, Kriging and response surface models are optimal methods to reduce time complexity for large problems. Prediction methods with multiple variables increase their computational complexity much faster than the growth of a dataset [21]. Moreover, it is a big challenge for the decision-maker to prioritise a specific set of data in extensive data for nonlinear prediction without losing any information [12].

Yager [22] introduced the OWA operator in a neural network to overcome the high dimensionality of a dataset. The OWA operator is the parameterised class of the mean type aggregation operator [23]. The approach reorders inputs before feeding them to the network. The process reduces input size significantly, consequently reducing the computational complexity. Building on the same concept, Cheng et al. [12] used the approach in the ANFIS model to handle a large dataset of the TAIEX stock index and predict future indexes. Bo et al. [24] used the same method by combining the IOWA layer with the Fruit Fly algorithm to predict vegetable price prediction. Although discussed, OWA approaches work well for different simple reordering and decision-making processes. However, the approaches cannot handle the complex reordering of input—QoS parameters in complex SLA management. In our recent experimental work [25–27], multiple OWA operators are combined with Analytic Hierarchy Process (AHP), Adaptive Neuro-Fuzzy Inference Systems (ANFIS) and different fuzzy clustering methods to accommodate the complexities of prediction data. The experimental results demonstrated high efficiency and better accuracy. The approach in [28] assists CSP selection by combining QoS and QoE.

To address the limitations of above discussed approaches, the paper uses the induced ordered weighted averaging (IOWA) layer in neural network structure. The distinctive features of the paper are as follows:

- The paper proposes a novel hybrid prediction model using the IOWA operator with multiple neural network methods for optimal QoS prediction.
- Existing QoS prediction approaches are unable to handle the complex relationships between parameters. The approach prioritises a specific data set from a big dataset for complex prediction.
- The method has the feature to reduce data size without losing any information to improve the complexity and retain accuracy.
- Unlike existing approaches, the approach can accommodate the custom requirements of the decision-makers for complex predictions.

To achieve above objectives, the paper combines the IOWA operator with nine neural network methods—Cascade-forward backpropagation (CFBP), Elman backpropagation (EBP), Feedforward backpropagation (FFBP), Generalised regression (GR), Nonlinear autoregressive exogenous (NARX), Layer recurrent neural network (LRNN), Long short-term memory (LSTM), Gated recurrent unit (GRU) and a combination of LSTM-GRU method. The paper analyses the prediction accuracy of the real cloud QoS dataset extracted from the Amazon EC2 US-West IaaS instance. Prediction accuracies are compared using a benchmark of RMSE, MAD and MAPE. The rest of the paper is organised as follows: Sect. 2 discusses related literature and preliminaries. Section 3 discusses the proposed approach. Section 4 demonstrates the evaluation results, and finally, Sect. 5 concludes the paper with future research directions.

## Preliminaries

This section discusses preliminaries and related studies that highlight the QoS prediction in a service-oriented environment.

### QoS prediction approaches

Several approaches have used various methods to predict QoS parameters. Smahi, Hadjila, Tibermacine and Benamar [29] proposed a Deep AutoEncoder (DAE)-based Matrix Factorization model for predicting the QoS of Web services. Gao et al. [30] applied a memory-augmented autoencoder for IoT time-series data. The model uses a clustering technique for input gathering to mitigate the data sparsity problem and enhance web QoS prediction accuracy. It also considers the influence of services/users' geographical characteristics to achieve accuracy [31]. Boutaba et al. [32] discuss the role of machine learning methods in QoS prediction. The survey highlights network management of traffic prediction, resource management, network security and QoS and QoE management. It also identifies parameters for QoS prediction and QoE factors to control the network-related problems. Rehman et al. [33] proposed a medical QoE (m-QoE) prediction model for ultrasound video streaming. The approach used Multilayer Perceptron Neural Network to extract device features to predict medical applications' QoS. Hussain et al. [34] analysed different soft computing approaches to predict QoS to form a viable SLA. Haytamy and Omara [3] proposed a Deep Learning-based Service Composition framework (DLSC). The approach assists cloud consumers to predict QoS-based services of cloud providers. The framework implements the LSTM deep learning method compounding with a Particle Swarm Optimization (PSO) algorithm. LSTM predicts the possible QoS values and is fed into the PSO, where the best service provider selection is made based on the resources required and minimised cost function of the consumer. Integration of Induced Ordered Weighted Average (IOWA), Weighted Average (WA), and Fuzzy time series are used to provide a novel prediction approach in the neural network framework [26]. The strategy has the advantage to manipulate difficult nonlinear predictions in the neural network architecture. Moreover, the technique also anticipates nonlinear statistical data. Using an ANFIS model, Harandizadeh et al. [35] created a novel hybrid intelligence system, ANFIS-PNN-ICA, that combined an adaptive neuro-fuzzy inference system (ANFIS) with a polynomial neural network (PNN), improved using the ICA algorithm i.e. Imperialism competitive algorithm for forecasting TBM performance. In another approach [27] authors proposed a unique clustered Induced Ordered Weighted Averaging (IOWA) Adaptive Neuro-Fuzzy Inference System (ANFIS), (CI-ANFIS) model. The approach used fuzzy time series prediction model to minimise data dimension and manages the cloud QoS nonlinear correlation. The approach incorporates a fuzzy neural network architecture for optimum forecasting results and an intelligent sorting method to handle prediction uncertainties.

Liu and Chen [36] defined two QoS prediction approaches in dynamic Cloud Manufacturing (CMfg). The personalised clustering approach uses textual and rating information to find the task similarity through a clustering algorithm. There is some probability of inaccuracy in predicting QoS values. However, the amalgamation of both prediction approaches has addressed the issue. Chen et al. [37] proposed a self-adaptive resource allocation framework that allows dynamic allocation of services on request. The process runs in an iterative feedback loop utilising an iterative QoS prediction model and a POS-based runtime decision algorithm. The model makes resource provisioning decisions based on iterations and repetitive feedback. The prediction model predicts QoS values in iterations, and the resultant value is then fed to the decision algorithm to find out the future automatic resource allocation procedures [38]. Liu and Chen [39] introduced a hybrid QoS prediction approach for dynamic cloud manufacturing prediction. The approach used a similarity enhanced collaborative filtering method for better prediction results and then applied a case-based reasoning method to better extract users and service details. The Bayesian function raises the accuracy of the proposed approach and reduces data uncertainty. To represent user-service interactions, Ma et al. [40] introduced a neural network-based framework called GCF (Generic Collaborative Filtering). The approach performs dropout regularisation to reduce the bias caused due to continuous values considered by QoS. It also decreases the high variance due to low-rank assumptions from a wide range of values.

Li et al. [41] formulated a Bayesian network model for cloud service prediction. First, the approach correlates the QoS parameters and hardware details from the infrastructure and platform layers. It then used a Bayesian network algorithm to predict future QoS parameters better. Hussain et al. [21] applied various neural network algorithms and compared them with stochastic methods to analyse the prediction accuracy at different intervals. Xu et al. [42] proposed a Neural Fusion Matrix Factorisation model for QoS predictions. The approach merged neural networks with the matrix factorisation technique to conduct nonlinear collaborative filtering for consumer and service latent selected features. Huang et al. [43] modelled an optimisation-based allocation mechanism in a cloud data centre depending on the user requirements. The approach initially assigns the arriving virtual machines that request from mobile devices. Users are then assigned to suitable physical machines depending on their hardware resource usage and the data centre's throughput status. CPU usage criteria are defined to determine which virtual machines are reassigned before and after allocation. Hussain et al. [25] presented a CQoES architecture for centralised Quality of Experience (QoE) and Quality of Service (QoS). The approach enabled cloud users in locating the best service provider by taking into account their top priorities. It also aided the service provider in intelligent resource management and decision-making for finite resources. The model used a combination of AHP, IOWA, POWA and Collaborative Filtering using KNN methods for evaluation that facilitates cloud stakeholders to establish a long-term, mutually beneficial relationship. Fu et al. [44] proposed a QoS prediction method using an improved nearest neighbour method for cloud service recommendation. The approach used the quantisation method to represent the stable status of services and users and then applied a NearestGraph method to get better prediction results. Keshavarzi et al. [45] proposed an enhanced time-aware QoS prediction method to avoid SLA violations in the cloud. The proposed approach employed a modified k-medoids algorithm to cluster data. The proposed approach addresses the cold start problem by using DTW Barycenter averaging algorithm. Zou et al. [46] proposed a neural network-based technique for temporal-aware service QoS prediction. The approach combined the binarisation facility and the similarity features for better temporal feature representation of users and services.

Deep learning models of Gated Recurrent Units (GRU) learn and extract temporal features across entities. Parameter optimisation is then used to train the DeepTSQP model to forecast undefined service QoS. Gao et al. [30] used the time series data of IoT sensors to predict the deviation in the system's behaviour and possible anomaly detection. The authors [47–49] applied different deep learning models such as LSTM, GRU and RNN to cloud QoS data. Alkalbani and Hussain [50] applied multiple machine learning methods such as SVM, KNN, Decision Tree and others to analyse cloud QoS data for optimal service discovery. Chowdhury et al. [51] proposed a QoS prediction model using Hybrid filtering and a Hierarchical prediction process. The hybrid filtering approach seeks to find a group of users and services similar to a target user. The hierarchical prediction process used hierarchical neural regression to forecast the QoS value properly. A comparative analysis of related approaches is presented in Table 1.

**Table 1:** Comparative analysis of existing approaches

Methods	Prediction method used	Manage nonlinear relationship	Manipulate high dimensional dataset	Handle variable QoS weights	Control complex reordering of decision making parameters	Data reduction without losing any information
Haytamy and Omara [3]	Deep Learning-based Service Composition framework (DLSC)	✓	✓	✗	✗	✗
Smahi et al. [29]	Deep AutoEncoder (DAE)-based Matrix Factorization model	✗	✗	✗	✗	✗
Liu and Chen [32]	Dynamic Cloud Manufacturing (CMfg) prediction approaches	✗	✓	✗	✗	✗
Chen et al. [33]	Self-adaptive resource allocation framework	✓	✗	✗	✗	✗
Li et al. [36]	Bayesian network model	✗	✗	✗	✗	✗
Fu et al. [37]	Novel nearest neighbour method for cloud service recommendation	✓	✓	✗	✗	✗
Keshavarzi et al. [38]	Enhanced time-aware QoS prediction model using K-medoids	✗	✓	✗	✗	✗
Ma et al. [40]	Generic Collaborative Filtering	✗	✗	✗	✗	✗
Xu et al. [42]	Neural Fusion Matrix Factorisation model	✗	✗	✗	✗	✗
Huang et al. [43]	Optimization-based allocation mechanism	✓	✓	✗	✗	✗
Zou et al. [46]	Neural network-based temporal-aware service QoS prediction model	✗	✗	✗	✗	✗
Chowdhury et al. [51]	Context-aware hierarchical QoS prediction with hybrid filtering	✓	✓	✗	✗	✗
Proposed CI-ANFIS	IOWA-ANFIS using minimax disparity approach with fuzzy c-means, subtractive clustering and grid partitioning	✓	✓	✓	✓	✓

The comparative analysis shows that although the above-discussed approaches forecast QoS parameters to help the decision-maker in the decision-making process. However, many drawbacks include managing nonlinear relationships, manipulating high dimensional datasets, and handling complex nonlinear predictions where different QoS parameters have additional weightage. Moreover, the approaches were unable to control the complex reordering of the decision-making parameters. They did not focus on aspect data reduction without any information loss. The proposed paper presents the IOWA layer in the neural network to address these shortcomings, as discussed in Sect. 3.

### OWA operator and families

The Ordered Weighted Averaging (OWA) operator introduced by Yager [23] is a family of mean-type operators. The OWA operator allows the aggregation realisation between the two extremes of OR and the AND [52, 53]. The operator is defined as follows:

#### Definition 1

The OWA operator of dimension n is a mapping OWA: R^n^ → R that has an associated weighting vector $W = \left( { w_{1} , w_{2} , w_{3} , \ldots \ldots , w_{n} } \right)$ such that w_i_ ϵ [0,1], i = 1,….., n and $\sum\nolimits_{i = 1}^{n} {w_{i} = 1}$. The operator is presented as:

1 $$OWA \left( { x_{1} , x_{2} , x_{3} , \ldots \ldots , x_{n} } \right) = \mathop \sum \limits_{i = 1}^{n} w_{i} y_{i}$$

where $\left( {y_{1} , y_{2} , y_{3} , \ldots , y_{n} } \right)$ is the reordered set of $\left( {x_{1} , x_{2} , x_{3} , \ldots , x_{n} } \right)$ from largest to smallest.

Another family of OWA operators is the Induced OWA (IOWA) operator. The IOWA operator [54] introduced by Yager and Filev is an aggregation operator that uses an induced variable to reorder input variables. The IOWA operator is defined as:

#### Definition 2

The IOWA operator of dimension *n* is a function *IOWA: R*^*n*^ → *R,* to which the weighting vector *W* of dimension *n,* $W = \left( { w_{1} , w_{2} , w_{3} , \ldots \ldots ., w_{n} } \right)$ is associated such that w_i_ ϵ [0,1], i = 1,….., n and $\sum\nolimits_{i = 1}^{n} {w_{i} = 1}$. It is defined to aggregate with the second set of arguments—induced variables $u_{i}$ such that:

2 $$IOWA \left( {u_{1} , j_{1} , u_{2} , j_{2} , \ldots \ldots , u_{n} , j_{n} } \right) = \mathop \sum \limits_{a = 1}^{n} w_{a} k_{a}$$

where $\left( { k_{1} , k_{2} , k_{3} , \ldots \ldots , k_{n} } \right)$ is the input argument $\left( { a_{1} , a_{2} , a_{3} , \ldots \ldots , a_{n} } \right)$ reordered based on an ordered inducing variable $\left( { u_{1} , u_{2} , u_{3} , \ldots \ldots , u_{n} } \right)$.

### Neural network prediction methods

This study analysed nine neural network methods to compare their prediction accuracy with the proposed approach. The methods are discussed as follows:

- *Feedforward backpropagation network:* A type of neural network also referred to as multi-layer perceptron that feedforward the values, calculate the error and propagate it back to the previous layer. The network comes with a hidden layer. Signals from the input layer are sent to neurons of the hidden layer in a weighted form which is further processed by the activation function. The output of each neuron is then sent to the output layer. The formulation of the network is presented as follows:
  3 $$O = f_{o} \left( {w_{b} + \mathop \sum \limits_{i = 1}^{k} w_{i}^{o} f_{h} \left( {w_{i}^{b} + \mathop \sum \limits_{j = 1}^{m} w_{ij}^{h} x_{j} } \right)} \right)$$
  where $f_{o}$, $f_{h}$ is the activation function in the output layer and hidden layer, respectively, ${w}_{b}$ is the weight from bias to output, ${w}_{i}^{b}$ represents a weight from bias to hidden layer.
- *Cascade-forward backpropagation network:* In this type of neural network, there is a connection from the input and every preceding layer to the subsequent layers. The method accommodates a nonlinear relationship between the input and the output. The formulation of the network is presented as follows:
  4 $$O = \mathop \sum \limits_{i = 0}^{n} f_{o} w_{i}^{i} x_{i} + f_{o} \left( {w_{b} + \mathop \sum \limits_{i = 1}^{k} w_{i}^{o} f_{h} \left( {w_{i}^{b} + \mathop \sum \limits_{j = 1}^{m} w_{ij}^{h} x_{j} } \right)} \right)$$
  where $w_{i}^{i}$ is the weight from the input layer to the output layer, $f_{o}$, $f_{h}$ is the activation function in the output layer and hidden layer, $w_{b}$ is the weight from bias to output and $w_{i}^{b}$ is the weight from bias to the hidden layer.
- *Elman backpropagation network*: This is a feedforward neural network with an extra layer of recurrent connection with tap delay. The network is comprised of four layers. The first and second layers are the input layer and hidden layer. The third layer is the undertake layer that memorises the hidden layer output, and finally, the fourth layer is the output layer. The formulation of the network is presented as follows:
  5 $$O = TF \left( {w_{Out} \times \left( {f \left( {w_{h} \times x \left( {k - 1} \right)} \right) + w_{In} \times u \left( {k - 1} \right)} \right)} \right)$$
  where *TF* is the transfer function, $w_{Out}$ is the weight of the hidden layer to the output layer, $x \left( {k - 1} \right)$ is the output of the undertaking layer, $w_{h}$ is the weight of the undertaking layer to the hidden layer, $w_{In}$ is the weight of the input layer to the hidden layer, $u \left( {k - 1} \right)$ is the input of neural network.
- *Generalised regression neural network*: This is a probabilistic neural network with a radial basis layer and a special linear layer. The method does not need the training process. Instead, it approximates the arbitrary function between input and output vectors. The approach is mostly used for function approximation. The generalised regression (GR) neural network comprises four layers: input, pattern, summation, and output. The formulation of the GR neural network is presented as follows [55]:
  6 $$O = \frac{{Sum_{s} }}{{Sum_{w} }}$$
  7 $$O = \frac{{\sum _{{j = 1}}^{n} \left( {\exp \left( { - \frac{{(in - a_{j} )^{t} (in - a_{j} )}}{{2\sigma ^{2} }}} \right)} \right)}}{{\sum _{{i = 1}}^{n} w_{i} p_{i} }}$$
  where $Sum_{w}$ is the weighted sum of the pattern layer outputs, $Sum_{s}$ is a simple summation of the pattern layer outputs, σ is a smoothing parameter, *in* is the input to the network, $a_{j}$ is the pattern vector for neuron *j*, *w* is the connection weight of a particular neuron to related neurons in the summation layer, and O is the network output.
- *NARX*: The nonlinear autoregressive exogenous (NARX) is a nonlinear autoregressive method that is widely used for time series prediction. The method is designed as a feedforward time-delay neural network that considers the same series of previous data. The method only takes the output neuron's feedback instead of the hidden neurons. The method can be mathematically represented as follows:
  8 $$O\left( {t + 1} \right) = f\left[ {o\left( t \right), \cdots , o \left( {t - d_{o} } \right);in\left( t \right), \ldots , in\left( {t - d_{in} } \right)} \right]$$
  9 $$O\left( {t + 1} \right) = f_{o} \left[ {b_{o} + \mathop \sum \limits_{k = 1}^{Nh} w_{ko} f_{k} \left( {b_{h} + \mathop \sum \limits_{j = 0}^{{d_{in} }} w_{jh} in\left( {t - j} \right) + \mathop \sum \limits_{i = 0}^{{d_{o} }} w_{ih} o\left( {t - i} \right)} \right)} \right]$$
  where *in(n), o(n)* are the input and output of the model at time interval *t*, $d_{in} \ge 1, d_{o} \ge 1$ is the input and output delay, $w_{jh} , w_{ko} , w_{ih}$ represents the weights of input, hidden, and output layers, $b_{h} , b_{o}$ are biases of hidden and output layers.
- *Layer recurrent neural network (LRNN)*: This is similar to a feedforward neural network excepting for recurrent connection with tap delay associated in each layer to have a finite dynamic response to the input dataset. The method is also widely used in different time series prediction analyses. The formula of the current RNN state is presented as:
  10 $$h_{t} = \tanh \left( {W_{inh} in_{t} + W_{hh} h_{t} + b_{h} } \right),$$
  11 $$O_{t} = W_{ho} h_{t} + b_{o}$$
  where, *in,o* represents input and output sequence, *h* represents hidden vector sequence at time interval *t*, *tanh is the* activation function used in the hidden layer, *W* represents weight matrices
- *LSTM:* The long short-term memory (LSTM) network is the extended version of the recurrent neural network developed by Hochreiter and Schmidhuber [56]. The standard recurrent neural networks cannot learn when the time lags are more than 5 – 10 distinct time steps between the observed and target data. The LSTM method overcomes the vanishing gradients and exploding gradients problems by introducing memory units or cell states. The typical formulation of a single LSTM cell is presented as follows [57]:
  12 $$In_{t} = \sigma \left( { W_{In} \times \left( {h_{t - 1} ,x_{t} } \right) + bi_{In} } \right),$$
  13 $$Fg_{t} = \sigma \left( { W_{Fg} \times \left( {h_{t - 1} ,x_{t} } \right) + bi_{Fg} } \right),$$
  14 $$Ou_{t} = \sigma \left( { W_{Ou} \times \left( {h_{t - 1} ,x_{t} } \right) + bi_{Ou} } \right),$$
  15 $$Cell_{t} = Fg_{t} \times Cell_{t - 1} + In_{t} \times \widetilde{{Cell_{t} }},$$
  16 $$\widetilde{{Cell_{t} }} = tnjh \left( { W_{Cell} \times \left( {h_{t - 1} ,x_{t} } \right) + bi_{Cell} } \right),$$
  17 $$h_{t} = Ou_{t} \times tnjh \left( {Cell_{t} } \right),$$
  where *W* represents weight matrix, *In* represents input gate, *Fg* represents forget gate, *Ou* represents output gate, *Cell* represents memory cell content, $\tilde{Cell }$ represents new memory cell content*, tnjh* represents a hyperbolic tangent function, σ represents a sigmoid function, *bi* represents biases, h represents hidden vector at time interval *t*, *x* is the input. The approach is commonly used in various time series prediction problems.
- *GRU*: The gated recurrent unit (GRU) is a similar network like LSTM, with a gated unit used to flow the information within the unit. It has fewer parameters than LSTM with no output gate and is more efficient than LSTM for the training process. The general formulation of the GRU network is presented as follows:
  18 $${\text{Re}}_{t} = sigf\left( { W_{{x{\text{Re}} }} x_{t} + W_{{y{\text{Re}} }} y_{t - 1} + bi_{{\text{Re}}} } \right),$$
  19 $$Up_{t} = sigf\left( { W_{xUp} x_{t} + W_{yUp} y_{t - 1} + bi_{Up} } \right),$$
  20 $$\tilde{y}_{t} = tnjh\left( { W_{xy} x_{t} + W_{yy} \left( {{\text{Re}}_{t} \odot y_{t - 1} } \right) + bi_{y} } \right),$$
  21 $$y_{t} = Up_{t} \odot y_{t - 1} + \left( { 1 - Up_{t} } \right) \odot \tilde{y}_{t} ,$$
  *Re* represents reset gate, *Up* represents update gate, *x,y* represents input and output vectors, *sigf* represents sigmoid activation function, *tnjh* represents a hyperbolic tangent function, *W* represents weight matrices, and *bi* represents biases.

## Proposed approach

This section introduces an IOWA layer in neural network structure to prioritise certain data for complex prediction [58]. The OWA operator enables aggregate information without losing any details from it. The proposed approach informs the prediction model that a certain set of data is of higher importance than the rest of the data that the existing methods cannot do. The approach uses the IOWA operator, where the weightage is assigned based on the inducing variable. The IOWA operator aggregates not only the numerical values but can accommodate objects as intervals, which enables the decision-maker to prioritise any particular set of data and make it ready for any complex predictions. This paper use cloud QoS data for complex QoS prediction. However, the approach can perform other complex predictions such as stock market, IoT sensor data, web service recommendation prediction and many others. The key feature of the approach is that it reduces the size of a dataset significantly without losing any information. This results in a reduction of computational time and complexity. The IOWA layer in a neural network is defined as follows:

### Definition 6

**Fig. 1:**
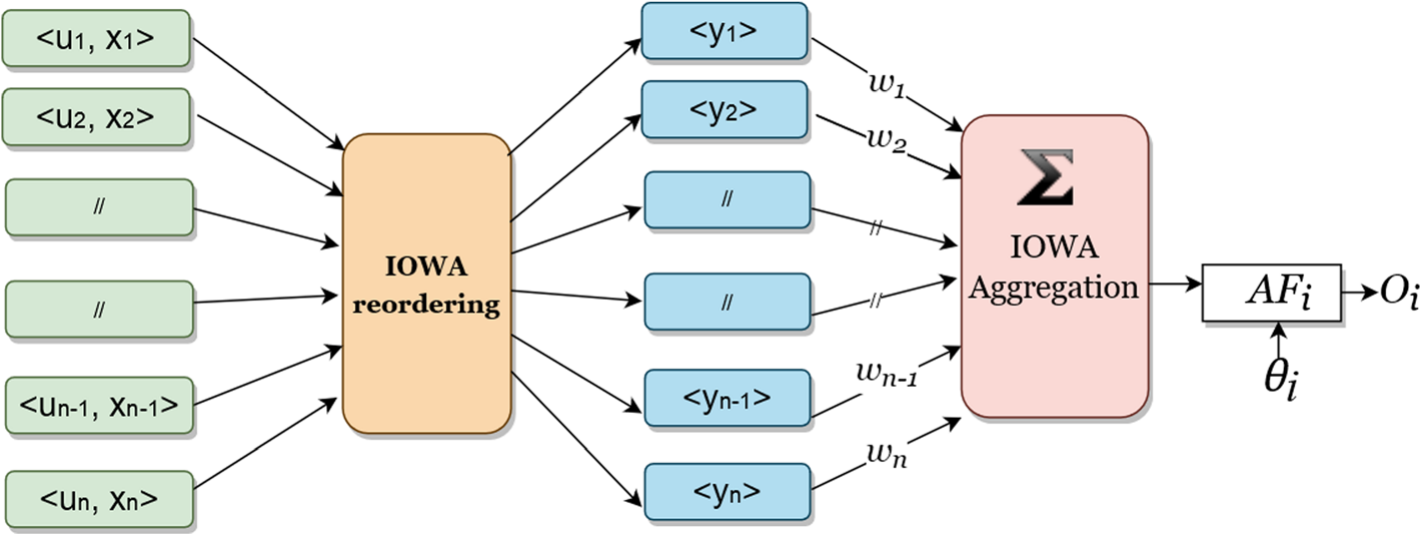
IOWA layer in neural network structure

The IOWA operator in neural network structure having an of inputs of *k* dimensions is a mapping IOWA: R^k^ → R defined by the associated weights *w* of dimension *k* such that w_i_ ϵ [0,1] and $\sum\nolimits_{i = 1}^{k} {w_{i} = 1}$ the set of inducing variables of order *ui,* as presented in Fig. 1 and Eqs. 22–23.

22 $$IOWA - NN \left( {u_{1} , x_{1} , u_{2} , x_{2} , \ldots \ldots ., u_{k} , x_{k} } \right) = AF_{i}$$

*AF*_*i*_ is the activation function which is the sum of the product of *w*_*i*_ and *b*_*i*_ which is

23 $$AF_{i} = \mathop \sum \limits_{i = 1}^{k} w_{i} y_{i}$$

where $\left\langle {u_{i} , x_{i} } \right\rangle$ is a set of two tuple input, where $u_{i}$ is inducing variable associated with the input $x_{i} , y_{i}$ is the reordered input $x_{i}$ in descending order of the $u_{i} , w_{i}$ is the associated $x_{i}$ weight, $O_{i}$ is the actual output of the output neuron.

The aggregated results then pass to the system, where it is compared with the threshold value $\theta_{i}$. The information is passed to the next layer neurons if the value is greater than or equal to the $\theta_{i}$. Otherwise, it drops the information as presented in the below equation:

24 $$AF_{i} \ge \theta_{i} , O_{i} > 0 \Rightarrow pass \vee AF_{i} < \theta_{i} ,O_{i} = 0 \Rightarrow drop$$

The paper considers an example where the decision-maker has time-series data and prioritises a certain data set without losing any information to predict the future interval to better understand the approach.

### Example

Let assume a decision maker has five set of input arguments with following values—*x* = (*x*_t_ = 50, *x*_t-1_ = 60, *x*_t-2_ = 20, *x*_t-3_ = 30, *x*_t-4_ = 50). The order of inducing variables for inputs are arranged as—*u* = (7, 2, 4, 5, 3). The paper considers following weights for each interval—*w* = (*w*_*1*_ = 0.25_,_ w_2_ = 0.10_,_ w_3_ = 0.30_,_ w_4_ = 0.15_,_ w_5_ = 0.20). The inputs are rearranged based on inducing variable are as follow *x* = (*x*_t_ = 50, *x*_t-3_ = 30, *x*_t-2_ = 20, *x*_t-4_ = 50, *x*_t-1_ = 60).

The activation function *AF* using Eq. 23 is calculated as:

- AF = [ (*w*_*1*_ × *x*_t_), (*w*_*2*_ × *x*_t-3_), (*w*_*3*_ × *x*_t-2_), (*w*_*4*_ × *x*_t-4_), (*w*_*5*_ × *x*_t-1_)].
- AF = [(0.25 × 50), (0.10 × 30), (0.30 × 20), (0.15 × 50), (0.20 × 60)].
- AF = 12.5 + 3 + 6 + 7.5 + 12.
- AF = 41.

## Implementation and evaluation

This section presents the performance and efficiency of the proposed approach and demonstrates the accuracy and improved computational complexity using a case study.

### Case study

To better understand the approach, the paper considers a complex scenario where the decision-maker prioritises certain data sets from large data to make a complex nonlinear prediction. The paper takes an example of cloud services where the decision-maker (service provider or a consumer) has a periodic record of QoS data. The decision-maker wants to prioritise certain data from the rest of the dataset for the custom requirements. The paper assumes that a decision-maker wants to analyse the QoS data of a cloud service for certain hours of a day. The decision-maker categorises the dataset into three working hours – peak hours, should hours and off-peak hours. Peak hours are those working hours when maximum activities of the business perform. In shoulder hours, some of the business activities are performed, while in off-peak hours, there are very few tasks that are performed.

Decision-maker categorises working hours as follows:

- Peak hours (PH): Let's assume that the decision-maker prioritises and define working hours from 9:00 AM to 5:00 PM as the peak hours. During these hours, the decision-maker executes their main task and rarely compromises on QoS variations.
- Shoulder hours (SH): Let's assume that the decision-maker takes two time periods for the shoulder period. The first period starts from 5:00:00 PM to 9:00:00 PM, and the second period starts from 5:00:00 AM to 9:00:00 AM.
- Off-peak hours (OH): Let's assume the decision-maker hours between 9:00:00 PM to 5:00:00 AM as off-peak hours.

The decision-maker is very concerned about the QoS behaviour during peak hours for the next interval, but at the same time, it also wants to consider the QoS data for all previous hours as well. The decision-maker prioritises 24 h as follows – PH < SH < OH. The symbol ‘<’ means precede in terms of priority and weightage.

### Experimental setup and dataset

The paper evaluates the approach in MATLAB R2020a, with a CPU of 1.8 GHz, RAM of 4.00 GB and storage of 1 TB. The Amazon EC2 US-West IaaS instance dataset is extracted from the PRTG monitoring service Paessler (www.paessler.com) for seven days from 20–04-2015 to 26–04-2015. The dataset comprised 5 min measurement of the cloud QoS data. Total of 2,016 records for training to predict the future eight hours are used. The traditional neural network used 2,016 records, and when applied the OWA neural network method, the records were reduced to 672 records for training to predict 96 intervals (peak 8 h) of the next day.

The approach evaluates EBP, FFBP, CCFBP, NARX, LR neural networks and their respective OWA methods with configuration settings. The approach uses a training function of TRAINLM, an adoption learning function of LEARNGDM with two layers. The first layer has 20 neurons, the transfer function for the hidden layer is TANSIG, and for the output layer, PURELIN is used. Training parameters are set as 1000 epochs with a maximum fail of 600. For LSTM and OWA- LSTM, the paper uses two LSTM layers, each with 100 units representing the dimension of the hidden state. The dropout value is set to be 0.5 with SIGMOID as an activation function and ADAM as an optimiser. The model runs through some 50 epochs. The GRU and OWA-GRU models contain three layers with 100 units each and a dropout value of 0.5. The activation function is LINEAR, and the optimiser is SGD. The number of epochs is set to be 50. The LSTM-GRU and respective OWA approaches consist of two LSTM and two GRU layers with a dropout of 0.5 each. The activation function is LINEAR, and the optimiser is ADAM.

### Evaluation

**Fig. 2:**
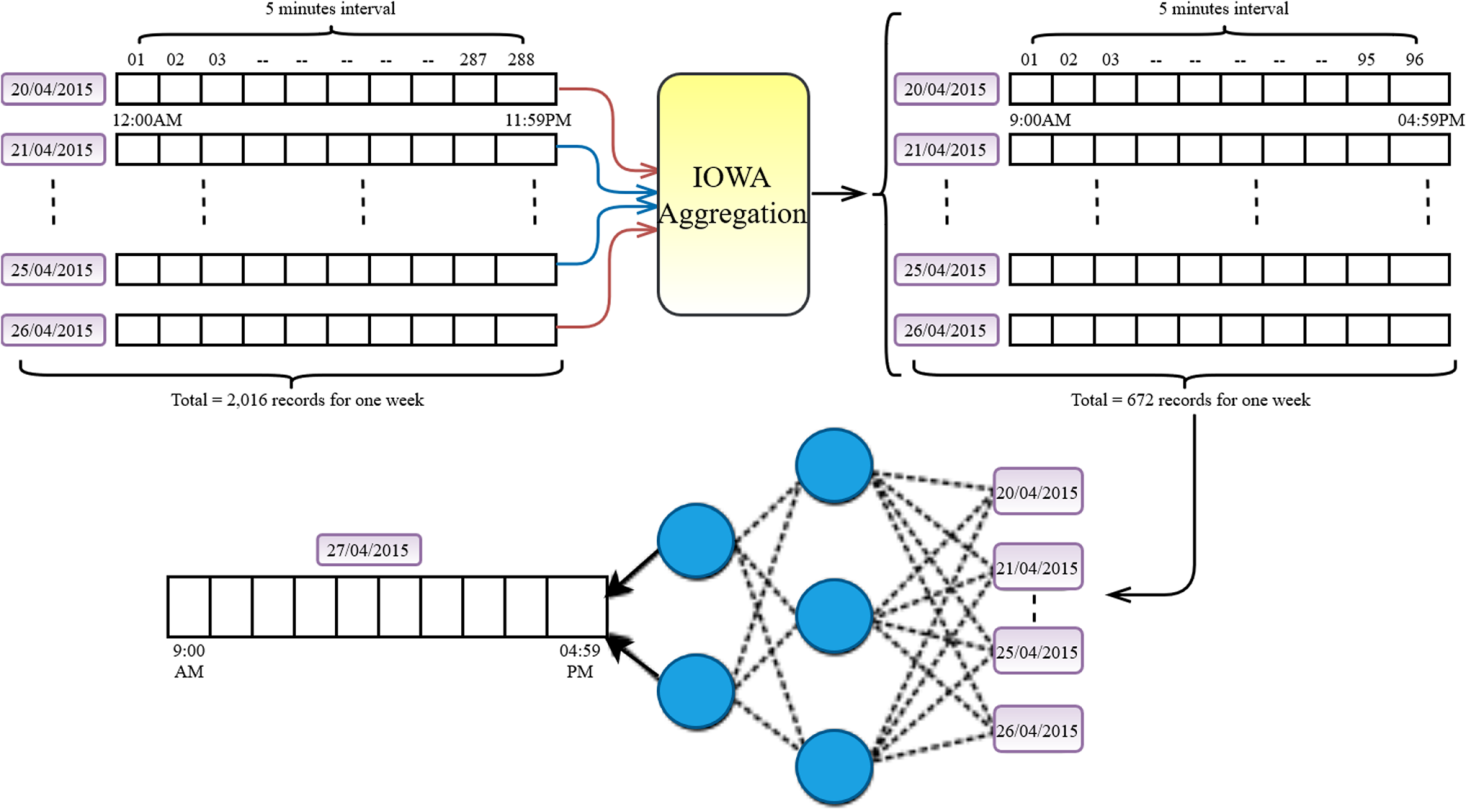
IOWA layer in prediction methods

The proposed approach works in two steps – IOWA aggregation and prediction, as presented in Fig. 2.

*IOWA aggregation*: Let assume the service provider have an optimistic behaviour therefore, the OWA weights are assigned as w1 = 0.55, w2 = 0.35 and w3 = 0.10. The paper considers the priority of time intervals as an inducing variable to OWA aggregation. The reordered intervals and working hours based on inducing variables are as below:

*u*_*1*_= PH = 9:00:00AM to 5:00:00 PM = 96 data intervals

*u*_*2*_= SH = 5:00:00PM to 9:00:00 PM , 5:00:00PM to 9:00:00 PM= 96 data intervals

*u*_*3*_= OH = 9:00:00PM to 5:00:00 AM = 96 data intervals

Applying Eq. (23), the paper gets the IOWA aggregated result for each day.

*Neural Network Prediction*: Let's consider nine neural network backpropagation algorithms for the experiment. For each of the approaches, the paper performs two sets of experiments. First, the paper predicts the QoS parameter for future peak hours, that is—27–04-2015, from 9:00:00AM to 5:00:00 PM using the default approach of the neural network method. The paper applies the proposed approach with the respective neural network method to predict future peak hours in the second experiment. The prediction accuracy of both approaches is measured using the following accuracy measurement benchmarks:

- *Root Mean Square Error (RMSE)*:
  RMSE is one of the most commonly used methods tomeasure prediction accuracy. It presents how far the prediction falls from the actual data using Euclidean distance. RMSE can be calculated using the square root of the mean of the square of all errors, as presented in the below equation.
  25 $$RMSE = \sqrt {\frac{{\mathop \sum \nolimits_{i = 1}^{N} \left( {O_{i} - P_{i} } \right)^{2} }}{N}}$$
  where *O* represents the observed data and *P* represents the predicted data.
  - *Mean Absolute Error (MAE)*
  MAE is another widely used metric to measure prediction accuracy. The method measures the average magnitude of the errors in a set of prediction results irrespective of their directions. MAE can be calculated by taking the average of absolute error, which is the absolute difference between the observed and predicted data where all individual differences have equal weights. MAE is presented in the below equation.
  26 $$MAE = \frac{1}{n} \times \mathop \sum \limits_{i = 1}^{n} \left| {O_{i} - P_{i} } \right|$$
  - *Mean Absolute Percentage Error (MAPE)*:
  MAPE is the average of the absolute percentage error of the predicted result. It gives the error result in terms of a percentage that makes it easier to understand. MAPE can be calculated as the mean absolute percentage error for each observed minus predicted divided by observed values. MAPE is presented in the below equation
  27 $$MAPE = 100* \frac{1}{N}\mathop \sum \limits_{k = 1}^{N} \left| {\frac{{O_{k} - P_{k} }}{{O_{k} }}} \right|$$

**Fig. 3:**
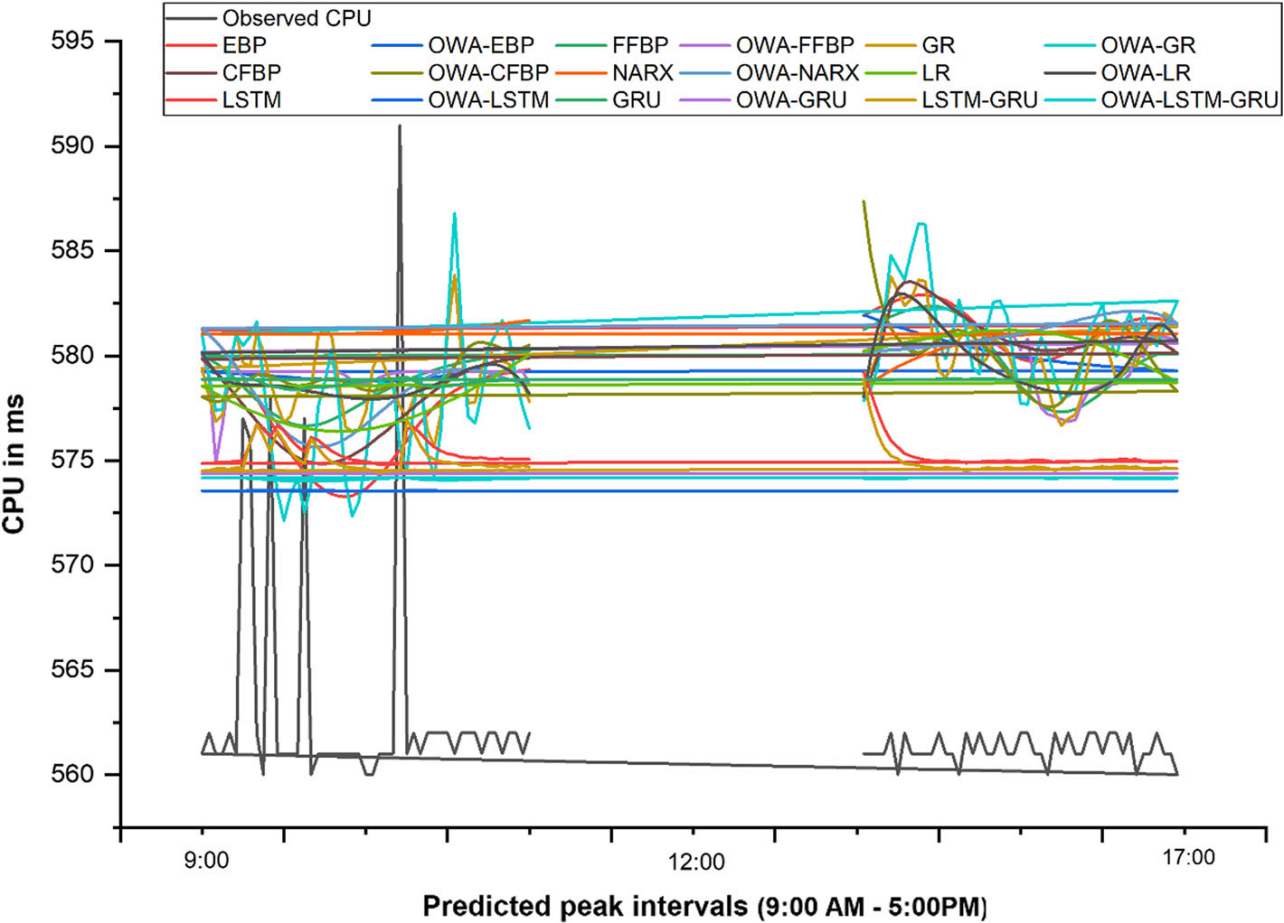
Prediction results of all methods

**Fig. 4:**
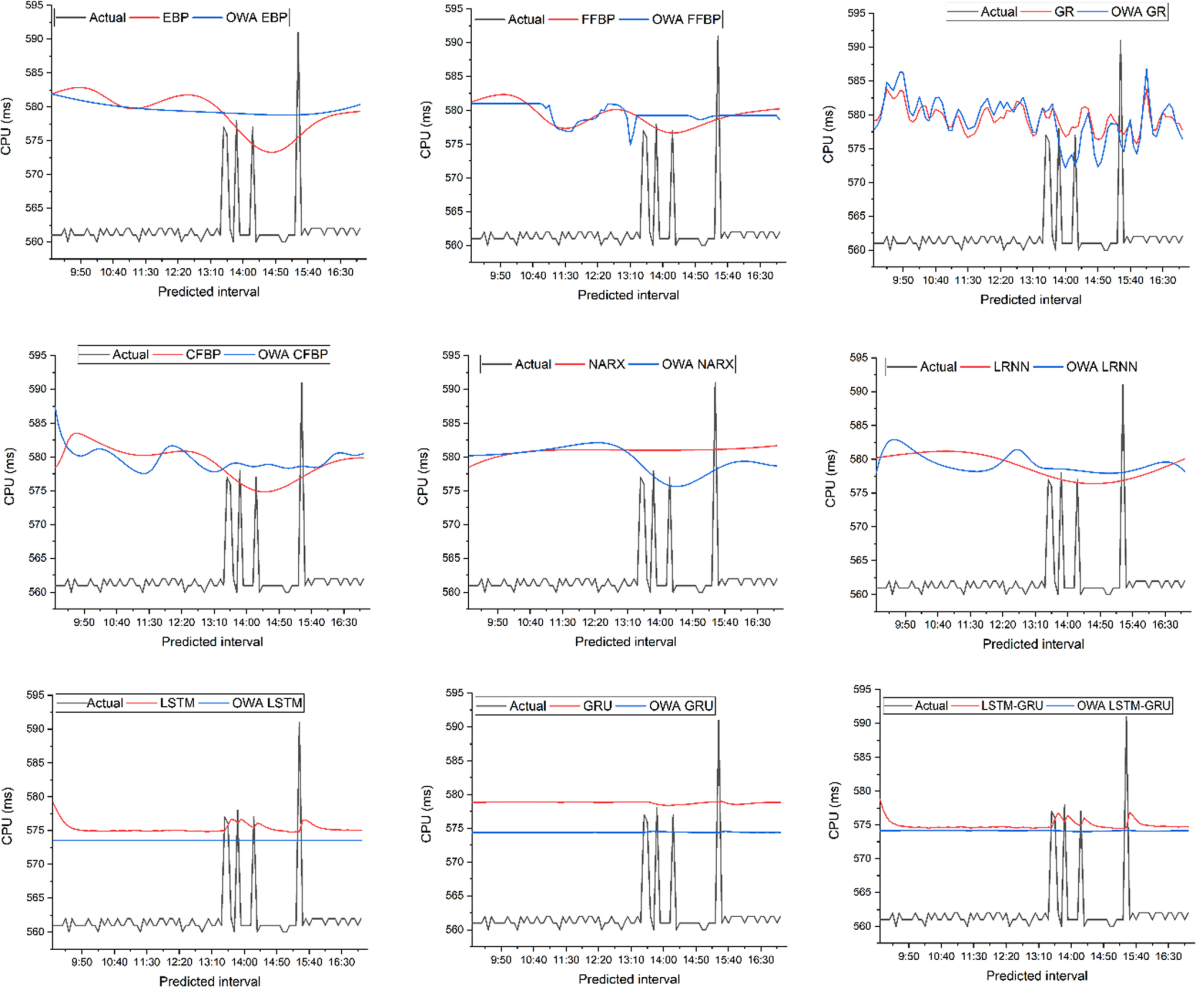
Neural network with respective OWA method

**Fig. 5:**
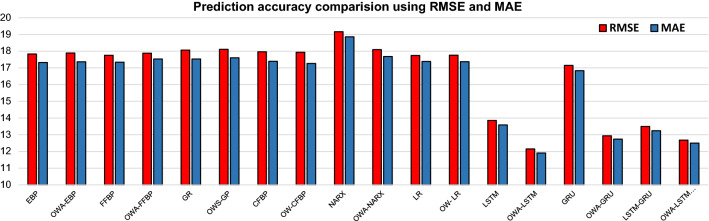
RMSE, MAE for all prediction methods

Table 2 presents the prediction results of each method for the first six hours. Figure 3 presents the predicted results of approaches for all intervals of peak hours. Figure 4 presents each neural network prediction behaviour with its respective OWA approach. The RMSE, MAE and MAPE of different methods are presented in Table 3 and Fig. 5.

**Table 2:** Prediction results of approaches for the next six hours

Date	Start time	End time	Obs. CPU	EBP	OWA-EBP	FFBP	OWA-FFBP	GR	OWA-GR	CFBP	OWA-CFBP
4/27/2015	9:00:00	9:05:00	561	581.909	581.942	581.240	581.010	579.224	577.863	578.452	587.366
4/27/2015	9:05:00	9:10:00	561	582.078	581.822	581.400	581.018	579.240	578.372	578.937	584.896
4/27/2015	9:10:00	9:15:00	561	582.241	581.704	581.558	581.018	580.058	579.398	579.881	583.197
4/27/2015	9:15:00	9:20:00	561	582.395	581.589	581.712	581.018	581.933	581.934	581.177	582.062
4/27/2015	9:20:00	9:25:00	562	582.536	581.478	581.858	581.018	583.777	584.806	582.396	581.299
4/27/2015	9:25:00	9:30:00	560	582.660	581.369	581.995	581.018	583.052	584.168	583.175	580.778
4/27/2015	9:30:00	9:35:00	562	582.762	581.264	582.116	581.018	582.369	583.625	583.499	580.428
4/27/2015	9:35:00	9:40:00	561	582.837	581.162	582.218	581.018	582.702	585.007	583.529	580.220
4/27/2015	9:40:00	9:45:00	561	582.879	581.062	582.296	581.018	583.610	586.303	583.403	580.157
4/27/2015	9:45:00	9:50:00	561	582.883	580.967	582.344	581.018	583.538	586.267	583.206	580.246
4/27/2015	9:50:00	9:55:00	561	582.844	580.874	582.356	581.018	581.218	583.357	582.976	580.472
4/27/2015	9:55:00	10:00:00	562	582.758	580.785	582.328	581.018	579.290	580.581	582.735	580.770
4/27/2015	10:00:00	10:05:00	561	582.623	580.699	582.253	581.018	578.980	579.843	582.491	581.039
4/27/2015	10:05:00	10:10:00	561	582.439	580.616	582.128	581.018	579.498	581.059	582.249	581.196
4/27/2015	10:10:00	10:15:00	560	582.210	580.537	581.949	581.018	580.788	582.668	582.013	581.212
4/27/2015	10:15:00	10:20:00	562	581.942	580.460	581.714	581.018	581.334	581.194	581.784	581.106
4/27/2015	10:20:00	10:25:00	561	581.644	580.387	581.425	581.018	581.330	579.426	581.566	580.908
4/27/2015	10:25:00	10:30:00	562	581.330	580.318	581.085	581.018	579.532	579.133	581.359	580.646
4/27/2015	10:30:00	10:35:00	561	581.013	580.251	580.702	581.018	579.866	581.264	581.165	580.336
4/27/2015	10:35:00	10:40:00	561	580.709	580.187	580.284	581.018	580.840	582.554	580.987	579.986
4/27/2015	10:40:00	10:45:00	562	580.431	580.127	579.847	581.018	580.582	582.618	580.825	579.604
4/27/2015	10:45:00	10:50:00	561	580.192	580.069	579.403	581.018	580.286	581.855	580.681	579.204
4/27/2015	10:50:00	10:55:00	561	579.999	580.014	578.969	580.914	579.271	579.802	580.556	578.809
4/27/2015	10:55:00	11:00:00	562	579.858	579.962	578.562	580.282	578.270	577.721	580.451	578.444
4/27/2015	11:00:00	11:05:00	562	579.770	579.913	578.197	580.781	578.270	577.683	580.368	578.125
4/27/2015	11:05:00	11:10:00	561	579.735	579.867	577.885	578.744	579.574	579.554	580.306	577.863
4/27/2015	11:10:00	11:15:00	561	579.749	579.822	577.637	577.513	579.894	580.880	580.266	577.667
4/27/2015	11:15:00	11:20:00	560	579.806	579.781	577.459	577.217	578.605	580.155	580.247	577.561
4/27/2015	11:20:00	11:25:00	562	579.901	579.741	577.354	577.093	577.271	578.928	580.249	577.583
4/27/2015	11:25:00	11:30:00	561	580.027	579.704	577.321	576.972	576.673	577.923	580.271	577.787
4/27/2015	11:30:00	11:35:00	562	580.177	579.669	577.356	576.850	576.983	578.100	580.310	578.227
4/27/2015	11:35:00	11:40:00	561	580.345	579.635	577.453	576.947	577.192	578.279	580.364	578.909
4/27/2015	11:40:00	11:45:00	561	580.523	579.604	577.605	577.745	578.127	579.644	580.431	579.749
4/27/2015	11:45:00	11:50:00	562	580.707	579.574	577.801	578.320	579.630	581.161	580.506	580.569
4/27/2015	11:50:00	11:55:00	562	580.890	579.546	578.033	578.504	580.766	581.782	580.586	581.199
4/27/2015	11:55:00	12:00:00	561	581.068	579.519	578.289	578.574	581.123	582.471	580.665	581.564
4/27/2015	12:00:00	12:05:00	562	581.236	579.493	578.560	578.619	580.035	580.836	580.740	581.683
4/27/2015	12:05:00	12:10:00	562	581.389	579.469	578.834	578.672	578.848	579.967	580.805	581.615
4/27/2015	12:10:00	12:15:00	561	581.523	579.445	579.103	578.769	579.195	581.138	580.853	581.419
4/27/2015	12:15:00	12:20:00	562	581.633	579.423	579.357	579.131	579.687	582.042	580.881	581.130
4/27/2015	12:20:00	12:25:00	560	581.716	579.401	579.586	580.063	579.501	580.974	580.881	580.771
4/27/2015	12:25:00	12:30:00	561	581.768	579.379	579.784	579.961	580.725	581.940	580.851	580.353
4/27/2015	12:30:00	12:35:00	561	581.785	579.358	579.943	580.920	580.518	581.062	580.785	579.897
4/27/2015	12:35:00	12:40:00	562	581.764	579.338	580.058	580.921	580.859	580.442	580.681	579.435
4/27/2015	12:40:00	12:45:00	561	581.701	579.317	580.123	580.890	582.047	581.297	580.535	579.003
4/27/2015	12:45:00	12:50:00	561	581.594	579.297	580.136	580.834	581.853	581.843	580.348	578.624
4/27/2015	12:50:00	12:55:00	560	581.440	579.277	580.095	580.571	581.375	582.615	580.119	578.308
4/27/2015	12:55:00	1:00:00	561	581.237	579.256	580.000	580.184	579.399	581.009	579.850	578.059
4/27/2015	1:00:00	1:05:00	562	580.984	579.236	579.853	578.945	578.054	579.031	579.545	577.885
4/27/2015	1:05:00	1:10:00	561	580.681	579.215	579.658	574.889	576.853	577.401	579.207	577.807
4/27/2015	1:10:00	1:15:00	561	580.329	579.194	579.420	576.704	577.189	577.445	578.843	577.851
4/27/2015	1:15:00	1:20:00	562	579.930	579.173	579.147	579.235	579.510	579.506	578.459	578.031
4/27/2015	1:20:00	1:25:00	561	579.489	579.152	578.849	579.264	581.029	581.006	578.063	578.326
4/27/2015	1:25:00	1:30:00	577	579.011	579.130	578.534	579.264	579.658	580.475	577.661	578.661
4/27/2015	1:30:00	1:35:00	576	578.502	579.108	578.214	579.264	579.539	580.924	577.262	578.938
4/27/2015	1:35:00	1:40:00	562	577.970	579.085	577.901	579.264	580.997	581.623	576.874	579.092
4/27/2015	1:40:00	1:45:00	560	577.426	579.063	577.605	579.264	580.211	578.793	576.505	579.115
4/27/2015	1:45:00	1:50:00	578	576.879	579.040	577.337	579.264	578.821	575.972	576.160	579.042
4/27/2015	1:50:00	1:55:00	561	576.339	579.017	577.105	579.264	577.671	573.520	575.848	578.913
4/27/2015	1:55:00	2:00:00	561	575.818	578.994	576.917	579.264	576.790	572.132	575.571	578.771

Date	Start time	End time	NARX	OWA-NARX	LR	OWA-LR	LSTM	OWA- LSTM	GRU	OWA-GRU	LSTM-GRU	OWA- LSTM—GRU
4/27/2015	9:00:00	9:05:00	578.467	580.196	580.234	579.219	573.560	578.871	574.389	578.863	574.153	579.219
4/27/2015	9:05:00	9:10:00	578.697	580.213	580.297	578.307	573.560	578.862	574.388	577.376	574.152	578.307
4/27/2015	9:10:00	9:15:00	578.914	580.231	580.360	577.327	573.560	578.858	574.384	576.264	574.152	577.327
4/27/2015	9:15:00	9:20:00	579.116	580.250	580.423	576.531	573.560	578.858	574.380	575.599	574.152	576.531
4/27/2015	9:20:00	9:25:00	579.306	580.271	580.486	575.958	573.560	578.862	574.376	575.218	574.153	575.958
4/27/2015	9:25:00	9:30:00	579.482	580.293	580.549	575.626	573.560	578.869	574.378	575.075	574.153	575.626
4/27/2015	9:30:00	9:35:00	579.645	580.317	580.611	575.323	573.560	578.870	574.375	574.837	574.154	575.323
4/27/2015	9:35:00	9:40:00	579.796	580.342	580.671	575.207	573.560	578.878	574.376	574.822	574.155	575.207
4/27/2015	9:40:00	9:45:00	579.936	580.369	580.730	575.109	573.560	578.880	574.377	574.749	574.156	575.109
4/27/2015	9:45:00	9:50:00	580.064	580.398	580.788	575.038	573.560	578.882	574.376	574.687	574.157	575.038
4/27/2015	9:50:00	9:55:00	580.181	580.428	580.843	574.990	573.560	578.885	574.375	574.643	574.158	574.990
4/27/2015	9:55:00	10:00:00	580.288	580.461	580.896	574.958	573.560	578.888	574.374	574.614	574.159	574.958
4/27/2015	10:00:00	10:05:00	580.386	580.496	580.945	574.991	573.560	578.892	574.378	574.675	574.159	574.991
4/27/2015	10:05:00	10:10:00	580.474	580.533	580.992	574.979	573.560	578.890	574.379	574.642	574.159	574.979
4/27/2015	10:10:00	10:15:00	580.554	580.572	581.035	574.958	573.560	578.887	574.378	574.610	574.159	574.958
4/27/2015	10:15:00	10:20:00	580.625	580.614	581.074	574.883	573.560	578.885	574.373	574.508	574.160	574.883
4/27/2015	10:20:00	10:25:00	580.690	580.659	581.109	574.921	573.560	578.892	574.374	574.595	574.161	574.921
4/27/2015	10:25:00	10:30:00	580.747	580.706	581.139	574.924	573.560	578.894	574.375	574.593	574.161	574.924
4/27/2015	10:30:00	10:35:00	580.798	580.756	581.163	574.974	573.560	578.895	574.379	574.661	574.161	574.974
4/27/2015	10:35:00	10:40:00	580.843	580.809	581.183	574.969	573.560	578.891	574.380	574.633	574.161	574.969
4/27/2015	10:40:00	10:45:00	580.883	580.864	581.196	574.952	573.560	578.887	574.379	574.604	574.160	574.952
4/27/2015	10:45:00	10:50:00	580.917	580.923	581.202	574.990	573.560	578.886	574.381	574.666	574.159	574.990
4/27/2015	10:50:00	10:55:00	580.947	580.985	581.202	574.978	573.560	578.881	574.381	574.637	574.158	574.978
4/27/2015	10:55:00	11:00:00	580.973	581.049	581.195	574.957	573.560	578.878	574.379	574.607	574.158	574.957
4/27/2015	11:00:00	11:05:00	580.995	581.117	581.180	574.993	573.560	578.878	574.381	574.669	574.157	574.993
4/27/2015	11:05:00	11:10:00	581.014	581.187	581.157	575.036	573.560	578.876	574.385	574.720	574.156	575.036
4/27/2015	11:10:00	11:15:00	581.030	581.261	581.126	575.016	573.560	578.869	574.385	574.670	574.155	575.016
4/27/2015	11:15:00	11:20:00	581.043	581.336	581.086	574.985	573.560	578.865	574.382	574.627	574.154	574.985
4/27/2015	11:20:00	11:25:00	581.053	581.414	581.038	574.901	573.560	578.864	574.375	574.520	574.154	574.901
4/27/2015	11:25:00	11:30:00	581.061	581.493	580.980	574.933	573.560	578.873	574.375	574.605	574.155	574.933
4/27/2015	11:30:00	11:35:00	581.068	581.573	580.914	574.932	573.560	578.878	574.375	574.602	574.156	574.932
4/27/2015	11:35:00	11:40:00	581.072	581.653	580.837	574.979	573.560	578.883	574.379	574.669	574.156	574.979
4/27/2015	11:40:00	11:45:00	581.075	581.732	580.752	574.973	573.560	578.881	574.380	574.639	574.156	574.973
4/27/2015	11:45:00	11:50:00	581.077	581.809	580.657	574.955	573.560	578.880	574.379	574.609	574.157	574.955
4/27/2015	11:50:00	11:55:00	581.078	581.883	580.553	574.992	573.560	578.881	574.381	574.670	574.156	574.992
4/27/2015	11:55:00	12:00:00	581.077	581.952	580.440	575.035	573.560	578.879	574.385	574.721	574.156	575.035
4/27/2015	12:00:00	12:05:00	581.076	582.013	580.317	575.016	573.560	578.872	574.385	574.671	574.155	575.016
4/27/2015	12:05:00	12:10:00	581.074	582.065	580.187	575.040	573.560	578.869	574.386	574.708	574.154	575.040
4/27/2015	12:10:00	12:15:00	581.072	582.104	580.048	575.070	573.560	578.865	574.389	574.743	574.152	575.070
4/27/2015	12:15:00	12:20:00	581.069	582.128	579.902	575.039	573.560	578.857	574.387	574.685	574.151	575.039
4/27/2015	12:20:00	12:25:00	581.065	582.132	579.749	575.055	573.560	578.855	574.387	574.717	574.150	575.055
4/27/2015	12:25:00	12:30:00	581.062	582.114	579.590	574.968	573.560	578.850	574.381	574.588	574.150	574.968
4/27/2015	12:30:00	12:35:00	581.058	582.068	579.425	574.929	573.560	578.855	574.376	574.566	574.150	574.929
4/27/2015	12:35:00	12:40:00	581.054	581.991	579.257	574.911	573.560	578.863	574.373	574.565	574.152	574.911
4/27/2015	12:40:00	12:45:00	581.050	581.878	579.084	574.957	573.560	578.874	574.376	574.646	574.153	574.957
4/27/2015	12:45:00	12:50:00	581.046	581.725	578.909	574.955	573.560	578.877	574.377	574.627	574.154	574.955
4/27/2015	12:50:00	12:55:00	581.042	581.530	578.733	574.942	573.560	578.880	574.377	574.603	574.155	574.942
4/27/2015	12:55:00	1:00:00	581.038	581.290	578.556	574.873	573.560	578.881	574.372	574.506	574.157	574.873
4/27/2015	1:00:00	1:05:00	581.034	581.004	578.380	574.860	573.560	578.889	574.369	574.516	574.159	574.860
4/27/2015	1:05:00	1:10:00	581.031	580.672	578.206	574.918	573.560	578.899	574.373	574.615	574.160	574.918
4/27/2015	1:10:00	1:15:00	581.028	580.298	578.034	574.928	573.560	578.900	574.375	574.608	574.161	574.928
4/27/2015	1:15:00	1:20:00	581.025	579.886	577.867	574.924	573.560	578.899	574.376	574.591	574.162	574.924
4/27/2015	1:20:00	1:25:00	581.022	579.444	577.705	574.972	573.560	578.899	574.379	574.659	574.162	574.972
4/27/2015	1:25:00	1:30:00	581.020	578.982	577.549	574.967	573.560	578.894	574.380	574.633	574.162	574.967
4/27/2015	1:30:00	1:35:00	581.018	578.510	577.400	575.838	573.561	578.908	574.441	575.882	574.154	575.838
4/27/2015	1:35:00	1:40:00	581.016	578.041	577.259	576.685	573.561	578.841	574.524	576.757	574.133	576.685
4/27/2015	1:40:00	1:45:00	581.015	577.588	577.127	576.570	573.561	578.686	574.544	576.159	574.107	576.570
4/27/2015	1:45:00	1:50:00	581.014	577.163	577.004	576.125	573.560	578.560	574.513	575.446	574.083	576.125
4/27/2015	1:50:00	1:55:00	581.014	576.776	576.891	576.681	573.561	578.522	574.532	576.423	574.058	576.681
4/27/2015	1:55:00	2:00:00	581.015	576.438	576.789	576.418	573.561	578.433	574.520	575.901	574.036	576.418

Obs. CPU: Observed CPU; OWA: Ordered Weighted Average; EBP: Elman backpropagation; FFBP: Feedforward backpropagation; GR: Generalised regression; CFBP: Cascade-forward backpropagation; NARX: Nonlinear linear autoregressive exogenous; LR: Layer recurrent; LSTM: Long short-term memory; GRU: Gated recurrent unit

**Table 3:** Prediction accuracy comparisons

	EBP	OWA-EBP	FFBP	OWA-FFBP	GR	OWS-GP	CFBP	OW-CFBP	NARX	OWA-NARX	LR	OW- LR	LSTM	OWA-LSTM	GRU	OWA-GRU	LSTM-GRU	OWA-LSTM GRU
RMSE	17.85	17.88	17.75	17.78	18.06	18.11	17.96	17.93	19.16	18.09	17.74	17.76	13.86	12.15	17.14	12.93	13.49	12.68
MAE	17.34	17.36	17.34	17.34	17.53	17.6	17.39	17.26	18.86	17.68	17.38	17.37	13.58	11.91	16.83	12.74	13.24	12.50
MAPE	3.10%	3.10%	3.10%	3.10%	3.10%	3.10%	3.10%	3.10%	3.40%	3.10%	3.10%	3.10%	2.40%	2.10%	3%	2.30%	2.40%	2.20%

**Fig. 6:**
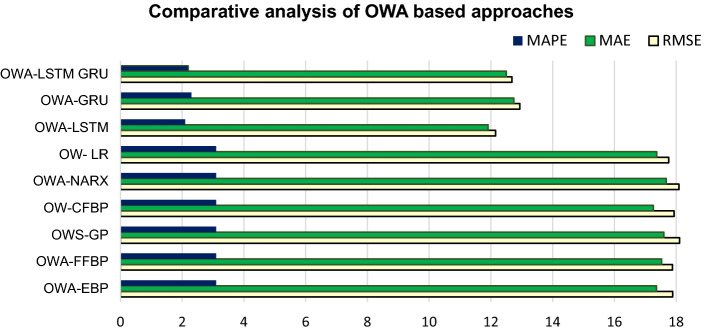
Comparative analysis of OWA approaches

The analysis result demonstrates that the approach significantly decreases the data size—by 66%, from 2016 to 672 records. From the prediction accuracy perspective, the proposed approach gives better or equal accuracy in almost all algorithms. There is a significant improvement in the GRU method when the approach includes the OWA layer. The RMSE has improved by 24%, from 17.144 to 12.937. The MAE has decreased from 16.83 to 12.74. The MAPE has decreased from 3 to 2.3%. Figure 6 presents a comparative overview of all OWA methods. The analysis result shows that OWA-LSTM gives the optimal prediction result to all other OWA methods with the RMSE of 12.15, MAE of 11.91 and MAPE of 2.10%.

## Conclusion

QoS prediction is one of the key factors to measure the quality of offered services. In a cloud environment, the agreed SLA is comprised of multiple offered services with several QoS parameters. The computational complexity of the system increases with the size of a dataset. Due widely spread of huge cloud QoS data, it is challenging to reduce the size of a dataset without losing any information. Existing approaches try to address the problem, but they cannot handle complex nonlinear predictions. The paper used the IOWA layer to predict nonlinear QoS prediction in the prediction method. The approach was tested using nine neural network methods, and their accuracies are compared with RMSE, MAE and MAPE. The experimental results demonstrate a notable data size reduction with better or equal prediction accuracy. The proposed method has significantly reduced the data size by about 66%, from 2,016 to 672 records, without losing any information. The GRU method has a significant improvement when the approach includes the OWA layer. The RMSE has improved by 24%, from 17.144 to 12.937. The MAE has decreased from 16.83 to 12.74. The MAPE has decreased from 3% to 2.3%. The experimental results evidenced that the approach handled complex nonlinear prediction by reducing data size with better or the same accuracies. In future, we will evaluate the approach to an extensive data IoT sensor network to make an informed decision.
